# Supplementary material for: Regulation of defective mitochondrial DNA accumulation and transmission in C. elegans by the programmed cell death and aging pathways
Source: eLife. 2023 Oct 2;12:e79725. doi: 10.7554/eLife.79725 (PMC10545429; doi:10.7554/eLife.79725)
Supplement: Supplementary file 1. [file elife-79725-supp1.docx]

**Supplementary Table 1:** A summary of all mutants analyzed in the PCD pathway, including their known homologs, whether they are part of the core PCD machinery, if they are pro-apoptotic or anti-apoptotic, whether they are mitochondrial proteins, and molecular details of the alleles analyzed.

| **Gene** | **Homolog** | **Core Machinery** | **Role in Apoptosis** | **Mitochondrial?** | **Allele** | **Parental Strain** | **Molecular Nature of the Allele** | **Protein Change** |
| --- | --- | --- | --- | --- | --- | --- | --- | --- |
| *ced-1* | SCARF2 | N | Pro(engulfment) | N | *e1735* | CB3203 | Substitution | Nonsense Q🡪Ochre |
| *ced-2* | CRK | N | Pro(engulfment) | N | *e1752* | CB3257 | Substitution | Nonsense W🡪Opal |
| *ced-3* | CASPASE | Y | Pro(executor caspase) | N | *n717* | MT1522 | Substitution | Splice site C🡪T |
|  |  |  |  |  | *n1286* | MT3002 | Substitution | Nonsense W🡪Opal |
|  |  |  |  |  | *n718* | MT1743 | Substitution | Missense G🡪R |
|  |  |  |  |  | *n2454* | MT8354 | Substitution | Missense A🡪T |
| *ced-4* | APAF1 | Y | Pro(apoptosome) | Y/N (primarily nuclear membrane) | *n1162* | MT2547 | Substitution | Nonsense Q🡪Ochre |
|  |  |  |  |  | *n1894* | MT5287 | Not curated | Unknown |
| *ced-9* | BCL2 | Y | Anti | Y | *n1950* | MT4770 | Substitution | Missense G🡪E |
| *ced-10* | RAC | N | Pro(engulfment) | N | *n1993* | MT5013 | Substitution | Missense V🡪G |
|  |  |  |  |  | *n3246* | MT9958 | Substitution | Missense G🡪R |
| *ced-13* | BH3 | N | Pro | N | *tm536* | FX536 | 523bp deletion | Only first 17bp remain |
|  |  |  |  |  | *sv32* | MD792 | 1304bp deletion | Complete knockout, also *inx-5* |
| *csp-1* | CASPASE | N | Pro(caspase) | N | *tm917* | JR3196 | 751bp deletion | Only first 59bp remain |
| *csp-2* | CASPASE | N | Anti | N | *tm3077* | JR3397 | 319bp deletion | Start at position 7317, deletes parts of exon 13 and all of 14 |
