# Supplementary material for: Regulation of defective mitochondrial DNA accumulation and transmission in C. elegans by the programmed cell death and aging pathways
Source: eLife. 2023 Oct 2;12:e79725. doi: 10.7554/eLife.79725 (PMC10545429; doi:10.7554/eLife.79725)
Supplement: Supplementary file 2. — (mean of 3 replicates ± standard deviation). [file elife-79725-supp2.docx]

**Supplementary Table 2.** Wildtype and mutant mtDNA levels in the different cell death and aging pathway mutants. (mean of 3 replicates ± standard deviation)

| **Nuclear Genotype** | **wildtype mtDNA copy number** | **uaDf5 mtDNA copy number** |
| --- | --- | --- |
| wild type | 1.5 x 10^5^ ± 2.4 x 10^4^ | 3.9 x 10^5^ ± 9.6 x 10^4^ |
| *ced-3(n717)* | 1.7 x 10^5^ ± 2.0 x 10^4^ | 5.7 x 10^5^ ± 9.9 x 10^4^ |
| *ced-3(n1286)* | 3.2 x 10^5^ ± 6.2 x 10^4^ | 14.8 x 10^5^ ± 2.6 x 10^5^ |
| *ced-3(n2454)* | 2.4 x 10^5^ ± 4 x 10^3^ | 6.7 x 10^5^ ± 2.5 x 10^4^ |
| *ced-3(n718)* | 3 x 10^5^ ± 2.5 x 10^4^ | 5.3 x 10^5^ ± 3.8 x 10^4^ |
|  |  |  |
| *csp-1(tm917)* | 1.7 x 10^5^ ± 5.3 x 10^3^ | 6.7 x 10^5^ ± 1.7 x 10^4^ |
| *csp-1(tm91); ced-3(n717)* | 2 x 10^5^ ± 2.2 x 10^4^ | 9.2 x 10^5^ ± 1.0 x 10^5^ |
| *csp-2(tm3077)* | 2 x 10^5^ ± 6.8 x 10^3^ | 5.7 x 10^5^ ± 8.5 x 10^4^ |
|  |  |  |
| *ced-13(sv32)* | 2.2 x 10^5^ ± 3.1 x 10^4^ | 9.1 x 10^5^ ± 2.0 x 10^5^ |
| *ced-13(tm536)* | 2.4 x 10^5^ ± 3.1 x 10^4^ | 12.2 x 10^5^ ± 2.2 x 10^5^ |
|  |  |  |
| *ced-4(n1162)* | 1.3 x 10^5^ ± 5.7 x 10^4^ | 4.1 x 10^5^ ± 2.0 x 10^5^ |
| *ced-4(n1894)* | 1.9 x 10^5^ ± 3.0 x 10^4^ | 6.2 x 10^5^ ± 1.0 x 10^5^ |
| *ced-9(n1950)* | 2.6 x 10^5^ ± 2.4 x 10^4^ | 5.7 x 10^5^ ± 5.0 x 10^4^ |
|  |  |  |
| *ced-1(e1735)* | 2.2 x 10^5^ ± 4.7 x 10^4^ | 9.3 x 10^5^ ± 2.3 x 10^4^ |
| *ced-2(e1752)* | 2 x 10^5^ ± 1.2 x 10^4^ | 9.0 x 10^5^ ± 1.2 x 10^5^ |
| *ced-1(e1735); ced-2(e1752)* | 1.2 x 10^5^ ± 3.0 x 10^4^ | 6.0 x 10^5^ ± 1.9 x 10^5^ |
| *ced-10(n1993)* | 1.1 x 10^5^ ± 6.3 x 10^3^ | 3.8 x 10^5^ ± 6 x 10^4^ |
| *ced-10(n3246)* | 1.8 x 10^5^ ± 2.3 x 10^4^ | 8.4 x 10^5^ ± 9.7 x 10^4^ |
|  |  |  |
| *daf-2(e1370)* | 4.08 x 10^5^ ± 4.8 x 10^4^ | 3x 10^5^ ± 4.8 x 10^4^ |
| *daf-2(e1391)* | 3.9 x 10^5^ ± 9.1 x 10^4^ | 1.1 x 10^5^ ± 3.2 x 10^4^ |
| *clk-1(qm30)* | 7.7 x 10^5^ ± 11.2 x 10^4^ | 2.3 x 10^5^ ± 1.9 x 10^4^ |
| *daf-2(e1391) clk-1(qm30)* | 3.5 x 10^5^ ± 3.5 x 10^4^ | 0 |
| *daf-16(mu86)* | 1.8 x 10^5^ ± 3.0 x 10^4^ | 6.1 x 10^5^ ± 1.3 x 10^4^ |
| *daf-16(mgDf50)* | 1.8 x 10^5^ ± 3.4 x 10^4^ | 6.2 x 10^5^ ± 1.1 x 10^4^ |
| *daf-16(mu86); daf-2(e1370)* | 2.4 x 10^5^ ± 1.8 x 10^4^ | 4.5 x 10^5^ ± 8.2 x 10^4^ |
| *daf-16(mu86); daf-2(e1391)* | 2.7 x 10^5^ ± 2.5 x 10^4^ | 2.8 x 10^5^ ± 2.4 x 10^4^ |
| *aak-2(ok524)* | 2.2 x 10^5^ ± 9 x 10^3^ | 8.5 x 10^5^ ± 1.0 x 10^4^ |
| *aak-2(gt33)* | 1.8 x 10^5^ ± 3.4 x 10^4^ | 6.2 x 10^5^ ± 1.1 x 10^4^ |
