# Supplementary material for: Regulation of defective mitochondrial DNA accumulation and transmission in C. elegans by the programmed cell death and aging pathways
Source: eLife. 2023 Oct 2;12:e79725. doi: 10.7554/eLife.79725 (PMC10545429; doi:10.7554/eLife.79725)
Supplement: Supplementary file 3. [file elife-79725-supp3.docx]

**Supplementary Table 3:** A summary of all lifespan mutants analyzed, including their known homologs, cellular pathways they are known to act in, whether the mutant extends or reduces lifespan, and molecular details of the alleles analyzed.

| **Gene** | **Homologs** | **Pathway** | **Allele** | **Parental Strain** | **Molecular Nature of the Allele** | **Protein Change** | **Mutant lifespan** | **Note** |
| --- | --- | --- | --- | --- | --- | --- | --- | --- |
| *aak-2* | AMPK | IIS pathway, adult lifespan, peptidyl-serine phosphorylation, regulation of protein localization | *gt33* | TG38 | 606bp deletion | Starts at position 3979, deletes exon 3 | Decrease | - |
|  |  |  | *ok524* | RB754 | 408bp deletion | Starts at position 4136, deletes part of exon 3 | Decrease | - |
| *clk-1* | COQ7 | Adult behavior, regulation of cellular metabolism | *qm30* | MQ130 | 590bp deletion | Starts at position 1044, deletes part of exon 4 and 5 | Increase | - |
| *daf-2* | IGFR | IIS pathway, cellular response to salt, negative regulator of macromolecule metabolic processes, positive regulation of developmental growth | *e1391* | DR1574 | Substitution | Missense P🡪L | Increase | ts |
|  |  |  | *e1370* | CB1370 | Substitution | Missense P🡪S | Increase | ts |
|  |  |  | *m41* | DR1564 | Substitution | Missense G🡪E | Increase | ts |
| *daf-16* | FOXO | IIS pathway, defense response to bacterium, regulation of cellular biosynthetic process, regulation of post-embryonic development | *mu86* | CF1038 | 1098bp deletion | Deletes 5 exons | Decrease | - |
|  |  |  | *mgDf50* | GR1307 | 20193bp deletion with TCTTCATTTTCAG insertion | Deletes 7 exons | Decrease | - |
| *fem-3* | novel | Male somatic sex determination, masculinization of hermaphrodite germline, positive regulation of macromolecule metabolic process | *q20* | JK816 | Not curated | Unknown | Increase | Gof and ts; female germline development inhibited |
| *glp-4* | VARS | Cell fate specification, embryonic pattern specification, positive regulation of cell proliferation | *bn2* | SS104 | Substitution | Missense G🡪D | Increase | ts; germline development inhibited |
