# Supplementary material for: Regulation of defective mitochondrial DNA accumulation and transmission in C. elegans by the programmed cell death and aging pathways
Source: eLife. 2023 Oct 2;12:e79725. doi: 10.7554/eLife.79725 (PMC10545429; doi:10.7554/eLife.79725)
Supplement: Supplementary file 4. [file elife-79725-supp4.docx]

**Supplementary Table 4:** *C. elegans* strains used in this study.

| **Strain** | **Genotype** | **Source** |
| --- | --- | --- |
| N2 | wildtype | CGC |
| LB138 | *him-8(e1489) IV; uaDf5/+* | CGC |
| JR3630 | *N2; uaDf5/+ (8x backcross version of LB138, him(e1489) was outcrossed)* | This study |
| JR3688 | *pink-1(tm1779); uaDf5/+* | This study |
| JR3880 | *ced-3(n717) IV; uaDf5/+* | This study |
| JR4330 | *csp-1(tm917) II; uaDf5/+* | This study |
| JR3977 | *ced-3(n1286) IV; uaDf5/+* | This study |
| JR3955 | *ced-13(sv32) X; uaDf5/+* | This study |
| JR3976 | *ced-13(tm536) X; uaDf5/+* | This study |
| JR3926 | *ced-10(n1993) IV; uaDf5/+* | This study |
| JR4001 | *ced-10(n3246) IV; uaDf5/+* | This study |
| JR3972 | *ced-1(e1735) I; uaDf5/+* | This study |
| JR3978 | *ced-2(e1752) IV; uaDf5/+* | This study |
| JR4010 | *ced-1(e1735)I; ced-2(e1752)IV; uaDf5/+* | This study |
| JR3938 | *ced-3(n2454) IV; uaDf5/+* | This study |
| JR3925 | *ced-4(n1162) III; uaDf5/+* | This study |
| JR4017 | *ced-4(n1894) III; uaDf5/+* | This study |
| JR3986 | *csp-2(tm3077) IV; uaDf5/+* | This study |
| JR4027 | *ced-9(n1950) III; uaDf5/+* | This study |
| JR3983 | *ced-3(n718) IV; uaDf5/+* | This study |
| MT1522 | *ced-3(n717) IV* | CGC |
| FX536 | *ced-13(tm536) X* | CGC |
| JR3966 | *glp-4(bn2) I; uaDf5/+* | This study |
| JR3949 | *fem-3(q20) IV; uaDf5/+* | This study |
| JR3937 | *atfs-1(et15) V; uaDf5/+* | This study |
| JR3960 | *daf-2(e1370) III; uaDf5/+* | This study |
| JR3963 | *daf-2(e1391) III; uaDf5/+* | This study |
| JR3993 | *clk-1(qm30) III; uaDf5/+* | This study |
| JR4060 | *daf-2(e1391) clk-1(qm30) III; uaDf5/+* | This study |
| JR3995 | *daf-16(mu86) I; uaDf5/+* | This study |
| JR3997 | *daf-16(mgDf50) I; uaDf5/+* | This study |
| JR4011 | *aak-2(ok524) X; uaDf5/+* | This study |
| JR4012 | *aak-2(gt33) X; uaDf5/+* | This study |
| JR4008 | *daf-16(mu86) I; daf-2(e1391) III; uaDf5/+* | This study |
| JR4005 | *daf-16(mu86) I; daf-2(e1370) III; uaDf5/+* | This study |
| JR3941 | *glp-1(q231) III; uaDf5/+* | This study |
| CF1038 | *daf-16(mu86) I* | CGC |
| MQ130 | *clk-1(qm30) III* | CGC |
| JR3981 | *csp-1(tm917) II; ced-3(n717) IV; uaDf5/+* | This study |
